# When to Account for Boundary Conditions in Estimating Hydraulic Properties from Head Observations?

**DOI:** 10.1111/gwat.12880

**Published:** 2019-04-02

**Authors:** L.J.M. Peeters, C. Turnadge

**Affiliations:** ^1^ CSIRO Land and Water, Locked Bag 2, Glen Osmond SA5064 Australia

## Introduction

Explicitly accounting for uncertainty in boundary conditions during calibration and uncertainty analysis invariably means increasing the complexity of a model. This increases computational requirements, as more parameters imply more model runs and more complex models often have a longer runtime. However, excluding these sources of uncertainty will lead to biased hydraulic property values and predictions, especially when only groundwater hydraulic head data are used in calibration (White et al. 2014).

In this technical commentary, we present a rule of thumb to help decide whether or not to account for uncertainty in boundary conditions when estimating hydraulic properties from groundwater hydraulic head observations. If the uncertainty in simulated hydraulic heads is dominated by uncertainty in hydraulic properties, boundary condition uncertainty will have a limited effect. Modeling efforts can then be aimed at refining the hydraulic property parameterization, such as characterizing spatial variability. On the other hand, if boundary condition uncertainty is the main contributor to hydraulic head uncertainty, it is very likely that hydraulic property estimates will compensate for incorrectly specified boundary conditions. In such conditions it is paramount to invest more in characterization of the boundary conditions (e.g., an elevation survey of a river network) and explicitly account for uncertainty in boundary conditions during parameter inference.

The next section details the derivation of the rule of thumb, which is then evaluated in the Results section for a wide range of hydrogeological settings. This is followed by a discussion on the potential implications for groundwater model development.

## Methods

**Figure 1:**
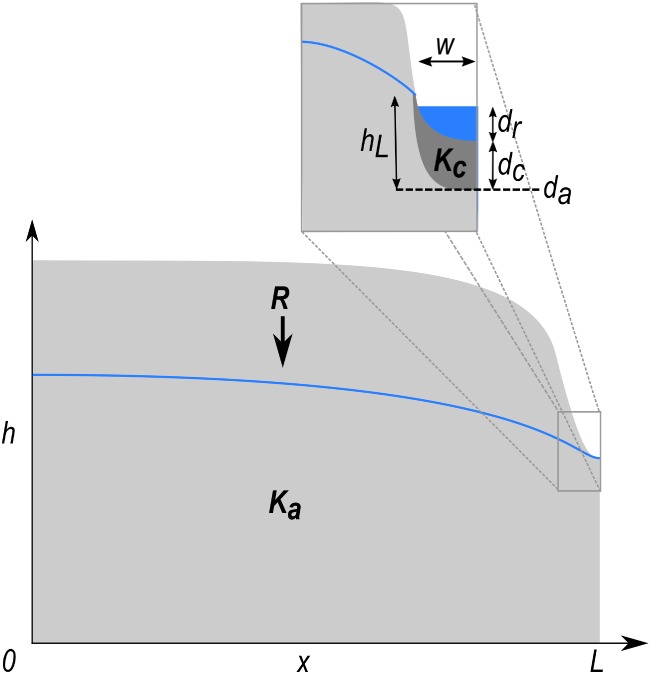
Conceptual cross section of an unconfined aquifer with uniform recharge *R* and hydraulic conductivity *K*_*a*_. A no‐flow (Neumann) boundary is located at *x* = 0 and a Cauchy boundary is located at *x* = *L*. The Cauchy boundary represents a river featuring a riverbed elevation *d*_*a*_, riverbed thickness *d*_*c*_, river stage *d*_*r*_, riverbed width *w* and hydraulic conductivity *K*_*c*_.

Groundwater hydraulic head *h*(*x*) in an unconfined aquifer featuring uniform recharge *R*, spatially uniform, hydraulic conductivity *K*_*a*_, a no flow boundary at *x* = 0 and a constant head boundary *d*_*a*_ + *h*_*L*_ at *x* = *L* (Figure 1), can be expressed as (Bear 1972, p. 380):

(1) $$h\left(x\right)=\sqrt{\frac{R}{K_{a}\;}\left(L^{2}-x^{2}\right)+{\left(d_{a}+h_{L}\right)}^{2}}$$

If the constant head boundary condition is replaced by a Cauchy boundary (e.g., representing a fully connected, partially penetrating river), then *h*_*L*_ will be in equilibrium with the river stage *d*_*r*_. The corresponding flux *Q* through a riverbed with width *w*, thickness *d*_*c*_, bottom elevation *d*_*a*_, and hydraulic conductivity *K*_*c*_ is:

(2) $$Q=-\frac{w\;K_{c}}{d_{c}}\left(d_{c}+d_{r}-h_{L}\right)$$

Under the steady state assumption that all recharge discharges to the river (*Q* = *RL*), Equation 2 can be written as a function of *h*_*L*_:

(3) $$h_{L}=\frac{R\;L\;d_{c}}{w\;K_{c}}+d_{c}+d_{r}$$

The simulated groundwater hydraulic head *h*(*x*) from Equation 1 will be most sensitive to boundary conditions when the contribution from the boundary condition is larger than the contribution from groundwater mounding; that is:

(4) $${\left(d_{a}+h_{L}\right)}^{2}>\frac{R}{K_{a}}\left(L^{2}-x^{2}\right)$$

which becomes, after combining with Equation 3 and reorganizing such that all boundary condition related terms are on the left hand side of the inequality:

(5) $$\frac{1}{R}{\left(\frac{R\;L\;d_{c}}{{\mathrm{wK}}_{c}}+d_{a}+d_{c}+d_{r}\right)}^{2}>\frac{\left(L^{2}-x^{2}\right)}{K_{a}}$$

This inequality can be used a simple rule of thumb to determine if groundwater level simulations in a study area are likely to be dominated by boundary conditions or by hydraulic properties.

Equation 5 can also be expressed as an index, as the log ratio of the left hand side and right hand side terms:

(6) $$\varepsilon =\log\left(\frac{\frac{1}{R}{\left(\frac{R\;L\;d_{c}}{{\mathrm{wK}}_{c}}+d_{a}+d_{c}+d_{r}\right)}^{2}}{\frac{\left(L^{2}-x^{2}\right)}{K_{a}}}\right)$$

Positive index values indicate when predicted hydraulic heads are most sensitive to boundary conditions. Conversely, negative index values indicate when predicted hydraulic heads are most sensitive to hydraulic properties.

## Results

The inequality in Equation 5 is straightforward to evaluate with values representative of a groundwater system to determine if boundary conditions are likely to dominate groundwater level simulations. It is however not trivial to establish from Equation 5 which factors are most influential or which combinations of factors are most likely to give rise to a situation in which hydraulic properties would dominate groundwater level simulations. To explore this in some more detail, we generated 10,000 samples using Latin Hypercube Sampling from a parameter space that we considered to be representative of a wide range of unconfined, shallow aquifer settings (Table 1). For the purpose of this analysis, we have made *x*, the position between the no flow and Cauchy boundary, dimensionless by dividing it by *L*, the distance between no flow and Cauchy boundary.

**Table 1:** Parameter Ranges Representative of Unconfined, Shallow Aquifers

Parameter	Description	Trans form	Minimum	Maximum	Units
*K*_*a*_	Aquifer hydraulic conductivity	log_10_	0.1	10	m/d
*K*_*c*_	Riverbed hydraulic conductivity	log_10_	0.01	1	m/d
*R*	Recharge	log_10_	2	200	mm/yr
*L*	Distance between no flow and Cauchy boundary	None	1000	10,000	m
*x*/*L*	Position between no flow and Cauchy boundary, as fraction of *L*	None	0.01	0.99	(−)
*d*_*c*_	Riverbed thickness	None	1	10	m
*d*_*r*_	River stage		1	10	m
*d*_*a*_	Aquifer thickness at Cauchy boundary	None	1	10	m
*w*	Riverbed width	None	1	10	m

**Figure 2:**
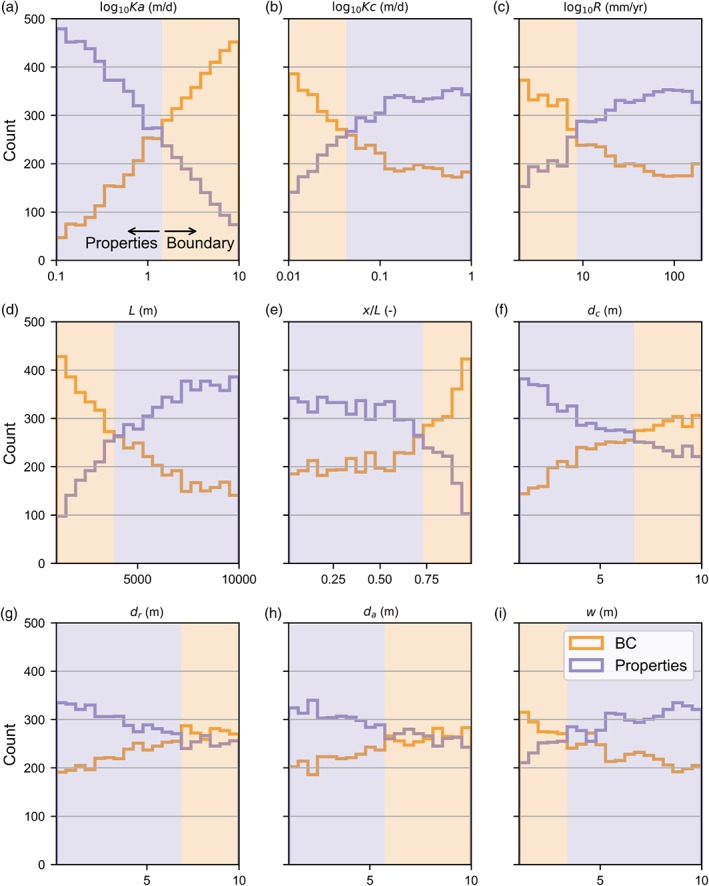
Histograms summarizing evaluations of the rule of thumb (Equation 5) with 10,000 parameter combinations, sampled uniformly using Latin Hypercube Sampling from parameter ranges listed in Table 1. Series labeled “Properties” represent parameter combinations for which hydraulic properties dominated the prediction of groundwater hydraulic heads at *x*/*L*, while series labeled “BC” indicate parameter combinations for which boundary conditions were dominant.

Figure 2 summarizes the results of evaluating all parameter combinations with histograms. For each parameter, the series labeled “Properties” show the parameter ranges for which hydraulic properties dominated the prediction of groundwater hydraulic head at *x*. Conversely, the series labeled “BC” show parameter ranges for which boundary conditions were dominant.

The most influential parameter was found to be aquifer hydraulic conductivity (*K*_*a*_) (Figure 2a). The hydraulic conductivity of the aquifer material will only dominate the groundwater level prediction for low values; less than 1 m/d in this example. This is quite intuitive as large hydraulic conductivities will result in small amounts of groundwater mounding. Similarly, the importance of boundary conditions becomes less important for larger recharge values (Figure 2c) as larger recharge results in greater groundwater mounding. Larger flow systems, with larger distances between no flow and river boundaries (e.g., more than 4 km), will be more prone to be dominated by hydraulic properties (Figure 2d). As the distance of the groundwater level prediction to the no flow boundary is a quadratic term in Equation 5, groundwater level predictions close to the Cauchy boundary will be dominated by boundary conditions, but at larger distances, hydraulic properties will dominate (Figure 2e). If we define riverbed conductance as the product of riverbed hydraulic conductivity and river width, divided by the riverbed thickness, it is clear that for low values of riverbed conductance (low *K*_*c*_ and *w*, high *d*_*c*_), boundary conditions are most likely to dominate (Figure 2b, 2i, 2f). The final component, aquifer thickness, has a less pronounced effect, although for smaller values, hydraulic properties are likely to dominate (Figure 2h). It has to be noted that in this formulation of the groundwater mounding equation (Equation 1), the bottom of the aquifer is the datum for the prediction of hydraulic heads. For hydraulic properties to dominate, the magnitude of groundwater mounding needs to be larger than the aquifer thickness below the river, which is obviously less likely for large aquifer thickness values.

## Discussion

The insights gained from the numerical exploration of the rule of thumb are as such not novel and one can even argue that these are almost trivial interpretations of groundwater flow equations as presented in most hydrogeology textbooks. For instance, rule 3.2 in Haitjema (1995, p. 28) states:

Rule 3.2: In regions where head‐specific boundary conditions are abundant, for example, many streams and lakes, the modeled heads are relatively insensitive to the choice of aquifer parameters (k and H), but are mostly determined by the heads specified at the surface waters.

However, like the heuristics presented in Haitjema (2006), Equation 5 can be very useful in (1) developing groundwater models, (2) undertaking groundwater model calibration and uncertainty analyses, and (3) prioritizing data collection.

Consider when Equation 5 indicates that hydraulic properties will dominate hydraulic head predictions. In this case, the heterogeneity of aquifer hydraulic properties is likely to affect predictions, and should be accounted for when developing a groundwater flow model. Historical hydraulic head observations are likely to be informative to constrain hydraulic properties. On the other hand, accounting for heterogeneity in riverbed conductance will probably be of secondary importance. Note that the potential for hydraulic properties to dominate is larger for hydraulic head predictions located further from the river boundary (Figure 2e). This result supports arguments for increasing weightings applied to observations located further from river boundaries in calibration and uncertainty analyses.

In the opposite case, where boundary conditions are likely to dominate groundwater level predictions, at least as much effort, if not more, should be directed at conceptualizing and parameterizing boundary conditions, as is invested in implementing hydraulic property heterogeneity, as argued by Voss (2011). It also indicates that during calibration, hydraulic properties inferred from historical observations are likely to be biased if boundary conditions are not correctly specified. In the same vein, predictive uncertainty will be underestimated if uncertainty in boundary conditions is not accounted for (Doherty and Welter 2010). This also means that relatively modest investments in the surveying of boundary condition elevations can result in greater reductions in predictive uncertainty than improved characterization of the heterogeneity of aquifer hydraulic conductivity.

The rule of thumb is intended as a screening tool and, due to its simplicity, it will not capture the full complexity of many groundwater systems. We caution against using the inequality in systems where the assumptions underpinning Equations 1, 2 are not satisfied, especially when (1) the system is not unconfined, (2) consists of multiple aquifers or perched aquifers, (3) the aquifer bed has a considerable slope, (4) there is strong spatial variability in recharge and hydraulic conductivity, and/or (5) other stresses strongly affect hydraulic heads, such as pumping or direct evaporation from the water table.

## Conclusion

Starting from the steady state groundwater flow equations describing groundwater mounding in an unconfined aquifer, a rule of thumb was developed that allows rapid assessment of whether hydraulic head predictions are likely to be dominated by hydraulic properties or by boundary conditions. The insights from this heuristic will hopefully be useful in achieving “elegant simplicity” in groundwater flow modeling (Schwartz et al. 2017). This may be achieved by focusing efforts in groundwater model design, calibration, uncertainty analysis, and data collection on those aspects that have the greatest potential to reduce predictive uncertainty.

## Conflict of Interest

None.
